# Simultaneous nodular lymphocyte-predominant Hodgkin lymphoma with papillary thyroid carcinoma: a case report

**DOI:** 10.1093/jscr/rjac599

**Published:** 2022-12-30

**Authors:** Daisuke Murayama, Toko Hashizume, Ryosuke Hirano, Koji Azuhata, Hisashi Shimojo, Nobuo Ito, Osamu Mishima

**Affiliations:** Department of Breast and Thyroid Surgery, Aizawa Hospital, Matsumoto, Nagano, Japan; Department of Breast and Thyroid Surgery, Aizawa Hospital, Matsumoto, Nagano, Japan; Department of Breast and Thyroid Surgery, Aizawa Hospital, Matsumoto, Nagano, Japan; Department of Pathology, Aizawa Hospital, Matsumoto, Nagano, Japan; Department of Pathology, Aizawa Hospital, Matsumoto, Nagano, Japan; Department of Pathology, Aizawa Hospital, Matsumoto, Nagano, Japan; Department of Breast and Thyroid Surgery, Aizawa Hospital, Matsumoto, Nagano, Japan

## Abstract

We herein report the case of a 48-year-old man diagnosed with nodular lymphocyte-predominant Hodgkin lymphoma (NLPHL, Stage IA) and papillary thyroid carcinoma (PTC, Stage I). Total thyroidectomy, left modified neck dissection and biopsy of the right cervical lymph node were performed. Postoperatively, NLPHL treatment was prioritized, and external radiation (30.6 Gy) was applied to the right neck. PTC was considered a high-risk category for recurrence due to extranodal invasion of lymph node metastasis, and radioactive iodine therapy (ablative dose, 1110 MBq) was administered. Both PTC and NLPHL showed no recurrence 18 months after surgery.

## INTRODUCTION

When lymph node enlargement is observed during preoperative examination for thyroid cancer, it is necessary to differentiate between thyroid cancer metastasis, reactive enlargement and malignant lymphoma. Here, we report a rare case of nodular lymphocyte-predominant Hodgkin lymphoma (NLPHL) with papillary thyroid carcinoma (PTC) and lymph node metastasis, which necessitated therapy.

## CASE PRESENTATION

**Figure 1:**
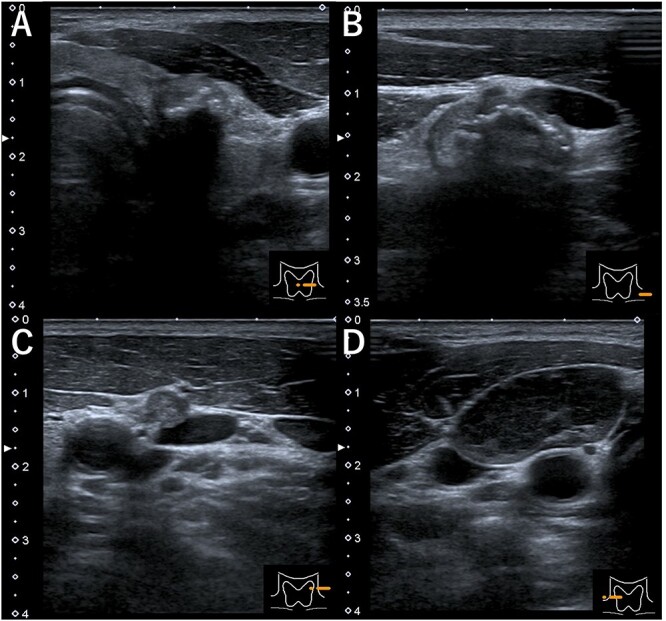
Ultrasonography (**A**) hypoechoic mass (18 × 14 mm) with microcalcification in the left lobe of the thyroid, (**B**) mass in the left cervical lymph node (#VI, 18 × 11 mm), (**C**) mass in the left cervical lymph node (#Va, 5 × 5 mm) and (**D**) the right cervical enlarged lymph node (#Vb, 24 × 12 mm), hypoechoic internally with no microcalcification.

**Figure 2:**
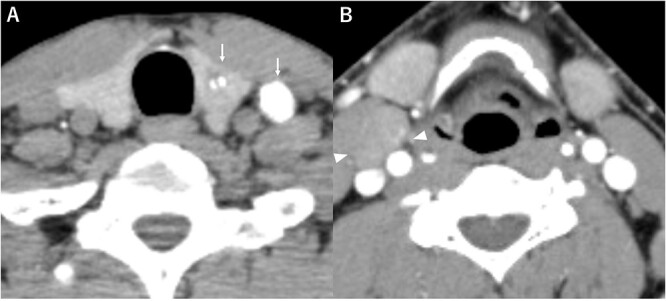
Computed tomography (**A**) calcified nodules in the left lobe of the thyroid and the left cervical lymph nodes (arrow), (**B**) the right cervical lymph node mass with no calcification or no contrast effect (arrowhead).

**Figure 3:**
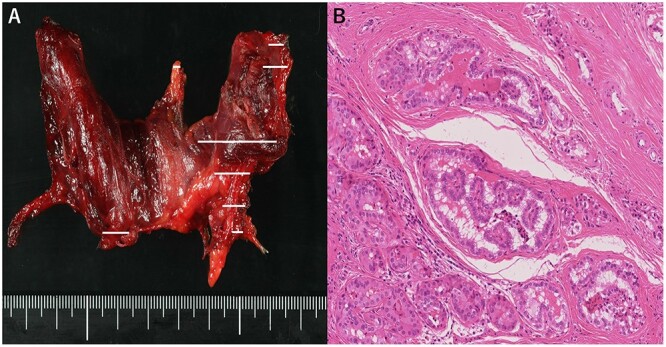
(**A**) Macroscopic aspect of the thyroid (tumor distribution: solid line), (**B**) Pathological diagnosis of papillary thyroid carcinoma. Hematoxylin and eosin staining, 200×.

**Figure 4:**
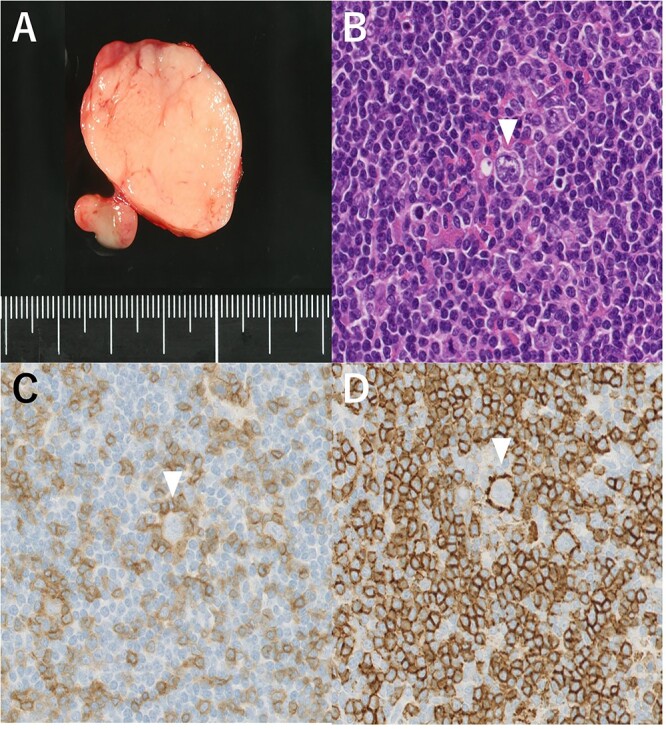
(**A**) Macroscopic aspect of the right cervical lymph node (#Vb). (**B**) Lymphocyte-predominant cells, termed “popcorn cells” (arrowhead) were observed against a background of B-cell-rich lymphoid follicles. Hematoxylin and eosin staining, 200×. (**C**) Immunostaining CD3 (–), 200×. (**D**) CD20 (+), 200×.

A 48-year-old man with a medical history of diabetes and pyorrhea alveolaris underwent computed tomography (CT). After the CT revealed a calcified mass in the left lobe of the thyroid and lymph nodes in the left cervical region, he was referred to our hospital. A solid mass was palpated in the left lobe of the thyroid and the left cervical lymph nodes. However, a soft mass was palpated in the right cervical region. He presented with no weight loss, no persistent fever and no night sweats (no B symptoms). Blood examination revealed that the patient was euthyroid, with a normal thyroglobulin level (17.4 ng/ml), a negative thyroglobulin antibody and a slightly high level of soluble interleukin-2 receptor (360 U/ml). Ultrasonography revealed a hypoechoic mass (18 × 14 mm) with microcalcification in the left lobe of the thyroid and a similar mass in the left cervical lymph node (#VI, 18 × 11 mm). The right cervical lymph node was enlarged (#Vb, 24 × 12 mm), although it was hypoechoic internally and had no microcalcification, differentiating it from the primary lesion (Fig. 1). CT revealed calcified nodules in the left lobe of the thyroid and the left cervical lymph nodes, but the right cervical lymph node mass was not calcified and showed no contrast effect (Fig. 2). Fine needle aspiration was conducted, and the mass in the left lobe of the thyroid was cytologically diagnosed as PTC. However, the right cervical lymph node mass remained class II. Thus, we preoperatively diagnosed PTC, cT1b N1b M0 cStage I and speculated that the right cervical lymph node mass expanded due to an unknown inflammation or malignant lymphoma. Total thyroidectomy, left modified neck dissection and biopsy of the right cervical lymph node were performed. The pathological diagnosis was PTC, 13 mm, T1b N1b (Fig. 3), with the right cervical lymph node diagnosed as NLPHL. Histologically, large tumor cells LP cells, termed “popcorn cells” were observed against a background of nodular or nodular diffuse proliferation of small lymphocytes. Immunostaining revealed that LP cells were CD3 (–), CD5 (–), CD10 (–), CD15 (–), CD20 (+), CD30 (–), CD79a (+), BCL6 (+) and EBER (–) in a background of B-cell-rich lymphoid follicles (Fig. 4). A postoperative fluorodeoxyglucose (FDG)-positron emission tomography (PET)/CT revealed no other accumulation except for the right cervical lymph node. The patient was diagnosed with Stage IA NLPHL, according to the Ann Arbor classification. Postoperatively, NLPHL treatment was prioritized, and external radiation (30.6 Gy) was applied to the right neck. PTC was considered a high-risk category for recurrence due to extranodal invasion of lymph node metastasis, and radioactive iodine therapy (ablative dose, 1110 MBq) was administered. Both PTC and NLPHL showed no recurrence 18 months after surgery.

## DISCUSSION

NLPHL accounts for 5% of all patients diagnosed with Hodgkin lymphoma (HL; [1]). The incidence of NLPHL has been reported in ~8–9 cases per 10 million people annually [2]. Binkley *et al*. reported a 5-year progression-free survival (PFS) rate of 87.1% and a 5-year overall survival (OS) rate of 98.3% [3]. Although the prognosis is favorable, large cell transformation can occur. In 10 years, 7% of NLPHL patients will have undergone transformation [4]. The prognosis of PTC is also favorable, with a 10-year OS rate of 97% reported [5].

Rizkallah *et al*. reported a case of HL presenting as typical B symptoms, which was incidentally diagnosed with PTC [6]. In addition, Liu *et al*. reported simultaneous HL and BRAF^V600E^-positive PTC and proposed that “lymphoma first approach,” and total thyroidectomy and RAI therapy for thyroid cancer, are necessary to improve the long-term survival of the synchronized condition [7].

In the pathological diagnosis of NLPHL, the lymph node architecture is totally or partially replaced by a nodular or mixed nodular and diffuse infiltrate composed primarily of small lymphocytes, macrophages and epithelioid histiocytes admixed with variable numbers of intermingled LP cells in a background of B-cell-rich lymphoid follicles [1]. LP cells are a key morphological feature of NLPHL and are called “popcorn cells” because their nucleus resembles an exploded popcorn kernel. LP cells are large and typically have a single large vesicular, polylobulated nucleus and distinct but small peripheral nucleoli, with no perinucleolar halos [8]. The presence of LP cells (popcorn cells) is required for NLPHL diagnosis. In terms of immunostaining, LP cells positively express CD20, CD79a, Pax5, Oct2 and Bob1 [9]. Unlike classical HL, LP cells stain negative for CD15 and CD30 [9]. Fan *et al*. identified six immunoarchitectural patterns of NLPHL (A: classic B-cell-rich nodular pattern, B: serpiginous nodular pattern, C: nodular pattern with many extranodal Langerhans cells, D: T-cell-rich nodular pattern, E: diffuse, T-cell-rich pattern, F: diffuse, moth-eaten B-cell-rich pattern). The E pattern predicts recurrence (*P* = 0.00324) [10]. Our case was an A pattern.

According to the International Lymphoma Radiation Oncology Group, radiotherapy (RT) was the primary treatment of NLPHL, and the 5-year PFS after RT (median dose of 36Gy) was 91.1% [3]. It was reported that the addition of chemotherapy did not improve outcomes [11].

Furthermore, Borchmann *et al*. reported that active surveillance for NLPHL was a viable initial management strategy because no significant differences were discovered in the 5-year OS between the active surveillance group and any treatment group (100% vs*.* 98%, *P* = 0.38) [12].

Our patient recovered without any postoperative complications, and external radiation could be performed promptly. When PTC is associated with malignant lymphoma, a reliable surgery without complications is required to avoid delay in the next treatment while aiming for adequate radial resection of PTC.

## CONCLUSION

We experienced a rare case of NLPHL with PTC. Since both tumors have a good prognosis, the best treatment should be selected considering the patient’s quality of life.

## Data Availability

Data related to our paper can be used as needed.

## References

[1] Swerdlow SH , Campo E, Harris NL, et al. (eds). WHO Classification of Tumours of Haematopoietic and Lymphoid Tissues. In: International Agency for Research on Cancer (IARC), revised 4th edn. Lyon, 2017, 431–4.

[2] Morton LM , Wang SS, Devesa SS, Hartge P, Weisenburger DD, Linet MS. Lymphoma incidence patterns by WHO subtype in the United States, 1992-2001. Blood. 2006;107:265–76. 16150940 10.1182/blood-2005-06-2508 PMC1895348

[3] Binkley MS , RaufMS, MilgromSA, PinnixCC, TsangR, DickinsonM, et al. Stage I-II nodular lymphocyte-predominant Hodgkin lymphoma: a multi-institutional study of adult patients by ILROG. Blood.2020;135:2365–74.3221187710.1182/blood.2019003877

[4] Al-Mansour M , Connors JM, Gascoyne RD, Skinnider B, Savage KJ. Transformation to aggressive lymphoma in nodular lymphocyte-predominant Hodgkin's lymphoma. J Clin Oncol. 2010;28:793–9. 20048177 10.1200/JCO.2009.24.9516

[5] Ito Y , Miyauchi A, Kihara M, Fukushima M, Higashiyama T, Miya A. Overall survival of papillary thyroid carcinoma patients: a single-institution long-term follow-up of 5897 patients. World J Surg. 2018;42:615–22. 29349484 10.1007/s00268-018-4479-z PMC5801380

[6] Rizkallah JJ , Jambart SS, Maalouli GD. Synchronous diagnosis of a Hodgkin lymphoma and a papillary carcinoma of the thyroid. Case Rep Intern Med 2014;1:235–7.

[7] Liu S , Zhao Y, Li M, Xi J, Shi B, Zhu H. Simultaneous Hodgkin lymphoma and BRAFV600E-positive papillary thyroid carcinoma: a case report. Medicine (Baltimore). 2019;98:e14180. 30653166 10.1097/MD.0000000000014180 PMC6370130

[8] Falini B , Bigerna B, Pasqualucci L, Fizzotti M, Martelli MF, Pileri S, et al. Distinctive expression pattern of the BCL-6 protein in nodular lymphocyte predominance Hodgkin's disease. Blood. 1996;87:465–71. 8555467

[9] Harris NL . Shades of gray between large B-cell lymphomas and Hodgkin lymphomas: differential diagnosis and biological implications. Mod Pathol. 2013;26:S57–70. 23281436 10.1038/modpathol.2012.182

[10] Fan Z , Natkunam Y, Bair E, Tibshirani R, Warnke RA. Characterization of variant patterns of nodular lymphocyte predominant hodgkin lymphoma with immunohistologic and clinical correlation. Am J Surg Pathol. 2003;27:1346–56. 14508396 10.1097/00000478-200310000-00007

[11] Ballas LK , Metzger ML, Milgrom SA, Advani R, Bakst RL, Dabaja BS, et al. Nodular lymphocyte predominant Hodgkin lymphoma: executive summary of the American radium society appropriate use criteria. Leuk Lymphoma. 2021;62:1057–65. 33274673 10.1080/10428194.2020.1852559

[12] Borchmann S , Joffe E, Moskowitz CH, Zelenetz AD, Noy A, Portlock CS, et al. Active surveillance for nodular lymphocyte-predominant Hodgkin lymphoma. Blood. 2019;133:2121–9. 30770396 10.1182/blood-2018-10-877761 PMC7022227

